# Telomere Length, Long-Term Black Carbon Exposure, and Cognitive Function in a Cohort of Older Men: The VA Normative Aging Study

**DOI:** 10.1289/EHP241

**Published:** 2016-06-03

**Authors:** Elena Colicino, Ander Wilson, Maria Chiara Frisardi, Diddier Prada, Melinda C. Power, Mirjam Hoxha, Laura Dioni, Avron Spiro, Pantel S. Vokonas, Marc G. Weisskopf, Joel D. Schwartz, Andrea A. Baccarelli

**Affiliations:** 1 Department of Environmental Health, and; 2 Department of Biostatistics, Harvard T.H. Chan School of Public Health, Boston, Massachusetts, USA; 3 Unidad de Investigación Biomédica en Cáncer, Instituto Nacional de Cancerología–Instituto de Investigaciones Biomédicas, Universidad Nacional Autónoma de México, Mexico City, Mexico; 4 Department of Epidemiology, Johns Hopkins Bloomberg School of Public Health, Baltimore, Maryland, USA; 5 Department of Clinical Sciences and Community Health, University of Milan, Milan, Italy; 6 Epidemiology Unit, Department of Preventive Medicine, Fondazione IRCCS Cà Granda Ospedale Maggiore Policlinico, Milan, Italy; 7 Veterans Affairs Boston Healthcare System, Boston, Massachusetts, USA; 8 Department of Epidemiology, Boston University School of Public Health, Boston, Massachusetts, USA

## Abstract

**Background:**

Long-term air pollution exposure has been associated with age-related cognitive impairment, possibly because of enhanced inflammation. Leukocytes with longer telomere length (TL) are more responsive to inflammatory stimuli, yet TL has not been evaluated in relation to air pollution and cognition.

**Objectives:**

We assessed whether TL modifies the association of 1-year exposure to black carbon (BC), a marker of traffic-related air pollution, with cognitive function in older men, and we examined whether this modification is independent of age and of C-reactive protein (CRP), a marker of inflammation.

**Methods:**

Between 1999 and 2007, we conducted 1–3 cognitive examinations of 428 older men in the Veterans Affairs (VA) Normative Aging Study. We used covariate-adjusted repeated-measure logistic regression to estimate associations of 1-year BC exposure with relative odds of being a low scorer (≤ 25) on the Mini-Mental State Examination (MMSE), which is a proxy of poor cognition. Confounders included age, CRP, and lifestyle and sociodemographic factors.

**Results:**

Each doubling in BC level was associated with 1.57 (95% CI: 1.20, 2.05) times higher odds of low MMSE scores. The BC-MMSE association was greater only among individuals with longer blood TL (5th quintile) (OR = 3.23; 95% CI: 1.37, 7.59; p = 0.04 for BC-by-TL-interaction). TL and CRP were associated neither with each other nor with MMSE. However, CRP modified the BC-MMSE relationship, with stronger associations only at higher CRP (5th quintile) and reference TL level (1st quintile) (OR = 2.68; 95% CI: 1.06, 6.79; p = 0.04 for BC-by-CRP-interaction).

**Conclusions:**

TL and CRP levels may help predict the impact of BC exposure on cognitive function in older men.

**Citation:**

Colicino E, Wilson A, Frisardi MC, Prada D, Power MC, Hoxha M, Dioni L, Spiro A III, Vokonas PS, Weisskopf MG, Schwartz JD, Baccarelli AA. 2017. Telomere length, long-term black carbon exposure, and cognitive function in a cohort of older men: the VA Normative Aging Study. Environ Health Perspect 125:76–81; http://dx.doi.org/10.1289/EHP241

## Introduction

Impaired cognition and dementia are leading causes of loss of independence in daily activities (McGuire et al. 2006), hospitalization (Chodosh et al. 2004), and mortality among older individuals (Bassuk et al. 2000; James et al. 2014). Consistent evidence has linked exposure to air pollution, specifically exposure to particulate matter, with poor age-related cognitive performance (Chen and Schwartz 2009; Power et al. 2011; Zeng et al. 2010) and accelerated cognitive decline (Weuve et al. 2012). In particular, exposure to air pollution particles from vehicular traffic, estimated from black carbon (BC) levels, has been associated with poor cognition in older men (Power et al. 2011). The limited availability of biomarkers to identify subsets of the general population that are at increased risk of age-related impaired cognition is a critical public health gap that hampers effective targeted prevention (Sperling et al. 2011). Although previous studies have identified a number of factors that may increase susceptibility to the effects of traffic air pollution on other health-related outcomes, such as cardiovascular or respiratory disease (Clougherty and Kubzansky 2009), no information is available at the present time to identify individuals who may suffer worse cognitive damage from air pollution exposure.

Telomeres are regions of repetitive DNA sequences at the ends of chromosomes that protect against DNA rearrangements and chromosomal end-to-end fusions and have established roles in biological aging (Blackburn 2001). Telomere length (TL) has been shown to decrease non-linearly with age across different tissues, including blood leukocytes, in living organisms (Saretzki and Von Zglinicki 2002; Young 2010). Shorter leukocyte TL has been associated with age-related diseases, such as cardiovascular disease (Fyhrquist et al. 2011), cancer (Artandi and DePinho 2010), and cognitive impairment (Roberts et al. 2014), as well as with mortality (Svenson et al. 2009), although one study reported opposing associations (Sanchez-Espiridion et al. 2014).

However, experimental data have shown that blood leukocytes carrying longer telomeres can build stronger inflammatory responses (Weng et al 1998). Indeed, cells with longer telomeres have greater capacity for rapid proliferation and clonal expansion (Hodes et al. 2002), which is critical for inflammatory cells to generate inflammatory responses. Inflammation is believed to play a central role in the effects of traffic air pollution, including those on cognitive function (Schram et al. 2007). Therefore, individuals with longer blood TL may be more susceptibile to the adverse cognitive effects of air pollution. To the best of our knowledge, however, whether individuals with longer blood TL have different susceptibility to BC exposure has never been investigated in relation to cognitive function.

In this study of older individuals participating in the U.S. Veterans Affairs (VA) Normative Aging Study (NAS), we measured blood TL, BC exposure, cognitive function, and C-reactive protein (CRP) levels. We examined whether the previously observed negative association of BC exposure with cognitive function (Power et al. 2011) was stronger in men with longer blood cell TL. To further clarify the role of inflammation, we also determined whether the association between BC exposure and cognition was stronger in men with higher levels of CRP, which is a marker of systemic inflammation.

## Methods

### Study Sample

The NAS is an ongoing longitudinal study of aging in men from eastern Massachusetts. The study was established in 1963 by the U.S. Department of Veterans Affairs (Bell et al. 1966). In total, 1,596 individuals free of chronic disease at recruitment were invited to undergo an in-person examination every 3–5 years since 1984. Participants provided information on medical history, lifestyle, and demographic factors and underwent a physical examination and laboratory tests at each visit. Starting in 1993, all participants underwent cognitive tests (Weisskopf et al. 2004). Collection of blood samples for molecular analysis, such as TL, began in January 1999 (Baccarelli et al. 2010; McCracken et al. 2010). We included in the present analyses all cognitive assessments (up to 3; average = 1.3 visits per participant) performed at the time of the first blood collection or later. Each study visit for which we had a cognitive assessment and information on TL, CRP, and confounding covariates was included in the study. Participants provided written informed consent at each visit, and the VA Boston Healthcare System Institutional Review Board approved the study. Participants who had experienced a stroke before the first cognitive assessment on or after 1 January 1999 (3.21% of individuals) were excluded from the study. Out of the initial 814 active participants in the NAS between 1999 and 2007, that is to say, during the period in which TL measurements were performed, a total of 428 men with complete TL measurements, exposure assessments, cognitive testing, and covariate data were included in the analysis (see Table S1). Compared with the participants who were included in the analysis, those excluded were older [mean age ± standard deviation (SD) of 73.6 ± 6.6 vs. 74.3 ± 6.8 years, respectively, *p* = 0.05], but exposure BC levels, TL, and CRP levels were not significantly different (*p* = 0.18, 0.21 and 0.08, respectively).

### Cognitive Testing

At each visit, participants completed the Mini-Mental State Examination (MMSE), a validated global cognitive test to screen for dementia (Tombaugh and McIntyre 1992). The MMSE assesses several cognitive domains, such as orientation, immediate and short-term recall, attention and calculation, word finding, figure construction, reading and writing skills, and ability to follow a three-step command. The range of scores is 0–30; however, the maximum score in this study was 29 owing to exclusion of a county identification question because the general population in Massachusetts is not knowledgeable of counties (Weisskopf et al. 2004). Cognitive data were considered from study visits between 1999 and 2007.

### Exposure Assessment

BC exposure at each participant’s address was estimated using a validated spatio-temporal regression model for the greater Boston, Massachusetts, area that predicts daily BC levels starting on 1 January 1995. The model is based on data from 148 monitoring sites, daily BC concentrations at a central monitor, and predictors based on meteorological conditions (e.g., wind speed), measures of land use (e.g., traffic density), and other descriptors (e.g., day of the week) at each monitor location. The validation steps included checks on robustness, graphical convergence and goodness of fit of the model (Gryparis et al. 2007). BC estimates obtained from this model were considered a surrogate for individual exposure to traffic-related air pollution at residential addresses. Average exposure during the year before each visit was estimated by taking the average of the 365 daily BC estimates at the participant’s residential address before the date of each cognitive assessment, as reported previously (Power et al. 2011). These estimates were used as proxy measures of long-term BC exposure because of their high correlation with averages of BC over long time windows (see Table S2).

### Blood Measurements: Telomere Length and C-Reactive Protein

Seven milliliters of whole blood were collected in ethylenediamenetetraacetic acid (EDTA) tubes by venous phlebotomy. Buffy coat was obtained from the blood samples and stored at –20°C until DNA isolation was performed using a QiAmp DNA blood kit (Qiagen, Germantown, MD, USA). Leukocyte TL was measured by quantitative real-time polymerase chain reaction (qRT-PCR), as described below. Relative leukocyte TL was measured by determining the ratio of the telomere repeat (T) copy number to a single-copy gene (S) copy number (T:S ratio) in a given sample. Human beta-globin was used as the single copy gene. To control for plate effects, leukocyte TL was calculated as relative units, expressing the ratio between leukocyte TL in the test DNA and leukocyte TL in a DNA pool used to generate a standard curve in each PCR. The standard pool consisted of DNA from participants randomly selected from the NAS (50 ng per sample) and was used in each run to create a standard curve, which ranged from 20 to 0.25 ng/μL of pooled DNA. An eight-point standard curve ranging from 30 to 0.234 ng/μL and derived from serially diluted pooled DNA was included in each PCR plate so that relative quantities of T and S could be determined.

For each sample, we prepared a 25-μL mixture of DNA sample (2 ng/μL) and carrier *Escherichia coli* DNA (15 ng/μL) to increase PCR reproducibility. These mixtures were heated to 96°C for 10 min and then cooled to room temperature. PCR primer sets for T and S and the PCR mix composition have been described previously (Hou et al. 2009). Using a MICROLAB STARlet Robot (Hamilton Life Science Robotics, Bonaduz, Switzerland), we transferred 4 ng of DNA in a 5-μL reaction mix into 384-well plates. We performed PCR runs on a 7900HT Fast Real-Time PCR System (Applied Biosystems, Foster City, CA, USA). After amplification, product specificity was confirmed by dissociation curve analysis. We ran all samples in triplicate, and the average of three T measurements was divided by the average of three S measurements to calculate the average T:S ratio (Farzaneh-Far et al. 2008). To test the reproducibility of this method, we amplified T and S in 15 samples in triplicate on 3 consecutive days. The coefficient of variation for the average T:S ratio of samples analyzed over 3 consecutive days was 8.7%, similar to the reproducibility originally reported for this method (Cawthon 2002).

We analyzed high-sensitivity CRP in fasting blood samples in duplicate and in a single batch to avoid between-batch analytical variation. The performance of the assays was monitored using standard quality control procedures including the analysis of quality control samples in each batch. CRP was measured in serum by immunoturbidimetric assays and using a Hitachi 917 analyzer (Roche Diagnostics, Indianapolis, IN, USA) with reagents and calibrators from Denka Seiken (Niigata, Japan) (McCracken et al. 2010). The high-sensitivity CRP analysis was performed in the laboratory of N. Rifai (Boston Children’s Hospital, Boston, MA), and the run-to-run CVs, at different hs-CRP-concentrations (0.47–54.9 mg/L), varied between 6.4% and 2.9% (Rifai et al. 1999).

### Statistical Analysis

We used SAS (v.9.2; SAS Institute Inc., Cary, NC, USA) for all analyses. The dependent variable was the MMSE score of each participant at each visit. This variable presented a ceiling effect because 15.43% of observations reached the maximum score of 29, and 9.65% of the scores were less than or equal to the typical screening cut-off score for dementia (MMSE = 24) (Weisskopf et al. 2004). To take into account this distribution, MMSE scores were dichotomized in all analyses as > 25 and ≤ 25. The ≤ 25 MMSE score category was considered low cognitive performance. We described the association among TL, chronological age, CRP, and BC concentration using Pearson’s correlations.

TL measurements, considered at each visit as a proxy of biological aging, were categorized into quintiles. This approach accommodates potential nonlinear associations. We estimated the main effects of both BC levels and TL on the odds of low MMSE scores (≤ 25) using logistic regression models with generalized estimating equations using independent correlation structure and empirical variance estimates to avoid bias and account for repeated BC levels and MMSE scores. Because the relationship between BC levels and the odds of low MMSE scores was log-linear (Power et al. 2011), we used natural log–transformed BC [ln(BC)] in all analyses:

ln(*p_ij_*/(1 – *p_ij_*)) = β_0_ + β_1_ × (ln(*BC_ij_*)) + β_2_ × X_2_ *_ij_* + … + β*_n_* × X*_nij_* (1)

where p is the proportion with low MMSE scores; β_0_ is the overall intercept; β_1_ is the regression coefficient representing the predicted ln-odds of a low MMSE score with a 1-unit increase in natural log–transformed BC concentration; β_2_ … β*_n_* are the regression coefficients for *n* covariates included in adjusted models; *i* = 1, 2,…, 428 represents the subject; and *j* = 1, 2, 3 represents the *j*th cognitive assessment.

To evaluate whether TL modified the association of BC exposure with cognitive function, we added to Equation 1 interaction terms for BC exposure and indicator terms for TL quintiles. We considered the lower-order TL term as the reference level. To determine whether the effect modification by TL quintiles was independent of the interaction of either age or CRP levels with BC levels, we fitted additional models that included two sets of interaction terms—either BC exposure and age or BC exposure and CRP levels—to the model with the interaction term for BC exposure and TL. Both age and CRP level were categorized by quintiles to facilitate interpretation and to evaluate nonlinear association.

All models were adjusted for potential confounders or predictors of cognitive function, including age at cognitive assessment (continuous), CRP level (continuous), and the following variables measured at baseline, such as education (< 12, 12–16, > 16 years), first language (English/not English), computer experience (yes/no), physical activity (< 12, 12–30, ≥ 30 metabolic equivalent hours/week), body mass index (< 25, ≥ 25 kg/m^2^), dark-meat fish consumption (< once/week, ≥ once/week), alcohol intake (< 2, ≥ 2 drinks/day), smoking status (never, current, former), percentage of the participant’s census tract that is nonwhite, percentage of the adult residents in the participant’s census tract with at least a college degree, whether the cognitive data were from the participant’s first cognitive assessment (yes/no), whether the participant was a part-time resident of the greater Boston area (yes/no), hypertension (yes/no), diabetes (yes/no), and coronary heart disease (CHD) (yes/no). Census variables were collected in 2000, and health conditions were diagnosed by physicians during each visit. Covariate selection was based on previous literature (Power et al. 2011) and on association with the outcome. Results were considered significant at *p* < 0.05.

We conducted a set of sensitivity analyses to confirm the robustness of our findings. We performed analyses excluding MMSE outliers, identified as continuous cognitive scores deviating three interquantile ranges from the first or third quartile, and observations with CRP levels indicative of an active immune response (CRP levels ≥ 10 mg/L) (Ridker 2003). In additional sensitivity analyses, we excluded hypertension, diabetes and CHD from the list of covariates because these variables may act as mediators of the BC–cognition relationship.

## Results

### Characteristics of Participants and BC Exposure Levels

Demographic and clinical characteristics of the cohort of 428 men at first cognitive assessment are shown in Table S3. Participants were 56–94 years of age with a mean ± SD age of 73.6 ± 6.6 years. Most individuals had at least some college or graduate-level education (71.03%), reported alcohol consumption of less than two drinks per day (78.27%), and were not affected by diabetes mellitus (82.24%) or CHD (70.79%) (see Table S3). The mean TL was 1.26 relative units (SD = 0.51) at the first visit, and TL was negatively correlated with age (Pearson’s *r* = –0.13, *p* = 0.01) and showed a decrease over subsequent visits (see Table S4). Of the 428 individuals included in this analysis, 21 had three cognitive assessments, 152 had two, and 255 had only one cognitive exam. The proportion of men with a low MMSE score increased from 18.7% at the first visit to 22.0% and 28.6% for men who completed second and third visits, respectively (see Table S4).

On the natural scale, 1-year average BC exposure estimates ranged between 0.02 and 1.90 μg/m^3^ (mean ± SD = 0.46 ± 0.23 μg/m^3^) and exhibited a right-skewed distribution. Because of this skewed distribution, we natural log–transformed BC exposure levels and reported associations for a doubling in BC concentration on the natural scale, or an approximately 0.69-unit change in ln(BC). BC exposure showed no significant correlation with age (Pearson’s *r* = –0.04, *p* = 0.41) or TL (Pearson’s *r* = –0.02, *p* = 0.62). Distributions of MMSE scores, BC levels, TL, and CRP measurements according to classes of age are shown in Table S4.

### BC Exposure, TL, CRP Levels, and Cognitive Function

BC exposure was significantly associated with higher relative odds of low MMSE scores (Table 1). A doubling of the average BC concentration during the previous year was associated with 1.57 times [95% confidence interval (CI): 1.20, 2.05] higher relative odds of low MMSE scores based on the covariate-adjusted model; this finding is consistent with, but not identical to (owing to moderately different sample size), previous estimates for this cohort (multivariable adjusted OR = 1.3; 95% CI: 1.1, 1.6 for a doubling of BC level during the previous year) (Power et al. 2011). TL, both continuous and categorized in quintiles, showed no significant association with low MMSE scores (Table 1). However, TL significantly modified the association between BC exposure and MMSE score. In particular, BC exposure had a significantly stronger association with MMSE scores in men with longer telomeres (5th quintile) (OR = 3.23; 95% CI: 1.37, 7.59; *p* = 0.04 for BC-TL interaction; Wald test: *p* = 0.03), whereas this association was null in men belonging to the other quintiles (Table 2).

**Table 1:** Relative odds of low Mini-Mental State Examination score (≤ 25)*^a^* associated with black carbon levels*^b^* and with telomere length, continuous and in quintiles.

Association with MMSE	OR (95% CI)	*p*	Cases (*n*)	Noncases (*n*)
BC concentration^*a*^^,^^*b*^	1.57 (1.20, 2.05)	0.001	124	498
TL (as continuous variable)^*a*^	0.97 (0.62, 1.52)	0.88	124	498
TL in quintiles^*a*^
TL 1st quintile (0.30–0.79)	Reference	–	28	96
TL 2nd quintile (0.80–1.04)	0.94 (0.52, 1.71)	0.84	25	99
TL 3rd quintile (1.05–1.25)	0.90 (0.46, 1.78)	0.77	22	103
TL 4th quintile (1.26–1.56)	1.33 (0.67, 2.66)	0.42	27	97
TL 5th quintile (1.57–3.81)	0.89 (0.43, 1.82)	0.75	22	103
Test for linear trend		0.79	124	498
Notes: BC, black carbon; 95% CI, 95% confidence interval; MMSE, Mini-Mental State Examination; OR, odds ratio; TL, telomere length. ^***a***^Adjusted for age (continuous), education level, first language, computer experience, physical activity level, body mass index, dark-meat fish consumption, alcohol consumption, smoking status, percentage of adults with a college degree, percentage of the participant’s census tract that is nonwhite, indicator for first cognitive assessment, indicator for part-time resident, hypertension, diabetes, coronary heart disease, and C-reactive protein levels (continuous). ^***b***^Association with each doubling in BC level, corresponding to a 0.69 μg/m^3^ increase in average ln(BC) concentration.				

**Table 2:** Modification by quintiles of telomere length of the relative odds of low Mini-Mental State Examination score (≤ 25)*^a^* associated with black carbon levels.*^b^*

Association between BC and low MMSE (≤ 25) by TL	OR (95% CI)	*p*	*p* for interaction^*c*^	Cases (*n*)	Noncases (*n*)
TL 1st quintile (0.30–0.79)	1.26 (0.83, 1.90)	0.27	–	28	96
TL 2nd quintile (0.80–1.04)	1.45 (0.96, 2.19)	0.08	0.62	25	99
TL 3rd quintile (1.05–1.25)	1.80 (0.89, 3.64)	0.10	0.39	22	103
TL 4th quintile (1.26–1.56)	1.05 (0.63, 1.75)	0.85	0.57	27	97
TL 5th quintile (1.57–3.81)	3.23 (1.37, 7.59)	0.01	0.04	22	103
All interaction terms at once (Wald test)			0.03	124	498
Notes: BC, black carbon; 95% CI, 95% confidence interval; MMSE, Mini-Mental State Examination; OR, odds ratio; TL, telomere length. ^***a***^Adjusted for age (continuous), education level, first language, computer experience, physical activity level, body mass index, dark-meat fish consumption, alcohol consumption, smoking status, percentage of adults with a college degree, percentage of the participant’s census tract that is nonwhite, indicator for first cognitive assessment, indicator for part-time resident, hypertension, diabetes, coronary heart disease, and C-reactive protein levels (continuous). ^***b***^Association with each doubling in BC level, corresponding to a 0.69 μg/m^3^ increase in average ln(BC) concentration. ^***c***^TL-by-BC level interaction.					

Because TL negatively correlates with chronological age, we also fitted models that included interaction terms between BC exposure and quintiles of age in addition to the interaction terms between BC exposure and TL quintiles. The MMSE-BC relationship was evaluated using two reference groups: the 5th age quintile and the 1st TL quintile. This model showed no significant interaction (*p* > 0.10) between age and BC exposure for any age group (Table 3). However, this model confirmed the interaction between TL and BC exposure and showed a substantially stronger association for BC exposure with relative odds of lower MMSE scores only among individuals with longer TL (5th quintile) and older age (5th quintile) (OR = 2.49; 95% CI: 1.07, 5.76; *p* = 0.04 for BC-by-TL interaction; Wald test: *p* = 0.13) than for those with shorter TL and older age (Table 3). In addition, a doubling of BC levels was associated with 2.49 times (95% CI: 1.07, 5.76) higher relative odds of low MMSE scores among men within the 5th TL quintile and the 5th age quintile. Only 17 participants (4%) were part-time residents of the greater Boston area, and their exclusion did not change the results significantly (data not shown).

**Table 3:** Modification by quintiles of telomere length and age of the relative odds of low Mini-Mental State Examination score (≤ 25)*^a^* associated with black carbon levels.*^b^*

Association between BC and low MMSE (≤ 25) by TL or age	OR (95% CI)	*p*	*p* for interaction	Cases (*n*)	Noncases (*n*)
TL 1st quintile (0.30–0.79)	1.07^*c*^ (0.56, 2.08)	0.84	–	28	96
TL 2nd quintile (0.80–1.04)	1.29^*c*^ (0.71, 2.35)	0.42	0.54^*e*^	25	99
TL 3rd quintile (1.05–1.25)	1.58^*c*^ (0.73, 3.45)	0.25	0.35^*e*^	22	103
TL 4th quintile (1.26–1.56)	0.92^*c*^ (0.42, 2.02)	0.84	0.67^*e*^	27	97
TL 5th quintile (1.57–3.81)	2.49^*c*^ (1.07, 5.76)	0.03	0.04^*e*^	22	103
Age 1st quintile (56–67)	1.46^*d*^ (0.51, 4.17)	0.49	0.54^*f*^	13	102
Age 2nd quintile (68–70)	1.59^*d*^ (0.51, 4.96)	0.44	0.43^*f*^	14	73
Age 3rd quintile (71–74)	0.93^*d*^ (0.40, 2.15)	0.87	0.62^*f*^	24	139
Age 4th quintile (75–78)	1.43^*d*^ (0.60, 3.41)	0.43	0.48^*f*^	19	90
Age 5th quintile (79–94)	1.07^*d*^ (0.56, 2.08)	0.84	–	54	94
All interaction terms at once (Wald test)			0.13	124	498
Notes: BC, black carbon; 95% CI, 95% confidence interval; MMSE, Mini-Mental State Examination; OR, odds ratio; TL, telomere length. ^***a***^Adjusted for education level, first language, computer experience, physical activity level, body mass index, dark-meat fish consumption, alcohol consumption, smoking status, percentage of adults with a college degree, percentage of the participant’s census tract that is nonwhite, indicator for first cognitive assessment, indicator for part-time resident, hypertension, diabetes, coronary heart disease, and C-reactive protein levels (continuous). ^***b***^Association with each doubling in BC level, corresponding to a 0.69 μg/m^3^ increase in average ln(BC) concentration. ^***c***^ORs for a doubling BC increase according to TL quintiles with Age 5th quintile as reference level. ^***d***^ORs for a doubling BC increase according to Age quintiles with TL 1st quintile as reference level. ^***e***^*p*-Values for TL-by-BC level interaction. ^***f***^*p*-Values for age-by-BC level interaction.					

To elucidate the role of inflammation in modifying the association between BC and cognitive function, we evaluated the influence of CRP levels on the association between BC exposure and MMSE scores. CRP levels were not correlated with TL (Pearson’s *r* = –0.01, *p* = 0.79), but they significantly modified the association of BC exposure with MMSE scores (Table 4). In this model, the MMSE-BC relationship was evaluated using the 1st CRP quintile and the 1st TL quintile as references. BC exposure had a stronger association with MMSE scores among participants with higher CRP levels (5th quintile) (OR = 2.68; 95% CI: 1.06, 6.79; *p* = 0.04 for BC-by-CRP interaction) (Table 4). BC exposure showed a substantially stronger association with relative odds of lower MMSE scores among individuals with longer TL (5th quintile) and lower CRP levels (1st quintile) (OR = 2.18; 95% CI: 0.77, 6.23; *p* = 0.03 for BC-by-TL interaction; Wald test *p* = 0.05) than among those with shorter TL and lower CRP levels (Table 4). CRP levels were not associated with MMSE scores (see Table S5). The BC-MMSE association did not show a monotonic pattern with quintiles of TL, age, and CRP.

**Table 4:** Modification by quintiles of telomere length and C-reactive protein of the relative odds of low Mini-Mental State Examination score (≤ 25)*^a^* associated with black-carbon levels.*^b^*

Association between BC and low MMSE (≤ 25) by TL or CRP	OR (95% CI)	*p*	*p* for interaction	Cases (*n*)	Noncases (*n*)
TL 1st quintile (0.30–0.79)	0.77^*c*^ (0.35, 1.71)	0.53	–	28	96
TL 2nd quintile (0.80–1.04)	1.07^*c*^ (0.44, 2.63)	0.89	0.34^*e*^	25	99
TL 3rd quintile (1.05–1.25)	1.28^*c*^ (0.48, 3.45)	0.64	0.32^*e*^	22	103
TL 4th quintile (1.26–1.56)	0.78^*c*^ (0.29, 2.09)	0.64	0.97^*e*^	27	97
TL 5th quintile (1.57–3.81)	2.18^*c*^ (0.77, 6.23)	0.14	0.03^*e*^	22	103
CRP 1st quintile (0.04–0.66)	0.77^*d*^ (0.35, 1.71)	0.53	–	24	99
CRP 2nd quintile (0.67–1.20)	1.54^*d*^ (0.72, 3.31)	0.27	0.16^*f*^	23	102
CRP 3rd quintile (1.21–2.04)	1.02^*d*^ (0.35, 2.96)	0.97	0.45^*f*^	32	92
CRP 4th quintile (2.05–3.99)	0.70^*d*^ (0.24, 2.04)	0.52	0.84^*f*^	24	100
CRP 5th quintile (4.00–72.20)	2.68^*d*^ (1.06, 6.79)	0.04	0.04^*f*^	21	105
All interaction terms at once (Wald test)			0.05	124	498
Notes: BC, black carbon; 95% CI, 95% confidence interval; CRP, C-reactive protein; MMSE, Mini-Mental State Examination; OR, odds ratio; TL, telomere length. ^***a***^Adjusted for age (continuous), education level, first language, computer experience, physical activity level, body mass index, dark-meat fish consumption, alcohol consumption, smoking status, percentage of adults with a college degree, percentage of the participant’s census tract that is nonwhite, indicator for first cognitive assessment, indicator for part-time resident, hypertension, diabetes, and coronary heart disease. ^***b***^Association for each doubling in BC level, corresponding to a 0.69 μg/m^3^ increase in average ln(BC) concentration. ^***c***^ORs for a doubling BC increase according to TL quintiles with CRP 1st quintile as reference level. ^***d***^ORs for a doubling BC increase according to CRP quintiles with TL 1st quintile as reference level. ^***e***^*p*-Value for TL-by-BC level interaction. ^***f***^*p*-Value for CRP level-by-BC level interaction.					

The BC-MMSE associations were similar to the main analyses when we excluded men with higher CRP levels and MMSE influential values and outliers (10 men) (see Tables S6–S8) and when we did not adjust for diabetes, hypertension, and CHD (see Tables S9–S11).

## Discussion

In a cohort of older urban residents, we observed that the association between 1-year exposure to traffic-related air pollution—as traced by time- and space-resolved estimates of BC levels—and global cognition may be augmented in individuals with longer leukocyte TL, even when adjusting for chronological age and CRP levels. We also showed that participants with higher CRP levels and in lower TL quintiles may exhibit stronger BC-MMSE associations. In addition, the BC-MMSE relationship did not show a monotonic pattern according to both TL and CRP quintiles. In the NAS cohort, BC level was previously associated with higher relative odds of impaired cognition (Power et al. 2011). Because we excluded participants without TL measurements, the estimates presented here are moderately different from those that were previously published (Power et al. 2011). Epidemiological studies have evaluated the association between air pollution and cognition in different populations, including children (Freire et al. 2010), adults (Chen and Schwartz 2009), and older individuals (Power et al. 2011; Ranft et al. 2009; Weuve et al. 2012). Those studies collectively suggest a negative impact of air pollution on cognitive function; however, whether biological factors, such as TL, age, and inflammation, modify this association had not been studied.

In human studies, particulate pollution has been shown to induce oxidative damage and inflammation (Araujo 2011), and BC-rich particles have been shown to cause both lung and systemic inflammation (Highwood and Kinnersley 2006). Inflammation has been extensively associated with dementia and cognitive decline in the elderly (Gorelick 2010). In addition, inflammatory molecules, such as cytokines, chemokines, and complement factors, have been found in cerebrospinal fluid and in beta-amyloid plaques in patients with Alzheimer disease (AD). Some evidence suggests that beta-amyloid and neurofibrillary tangles could provide inflammatory stimuli to microglia in AD (Gorelick 2010).

Leukocytes play a key role in inflammation because they coordinate all responses to antigen challenges in the human body (Granger and Senchenkova 2010). Our finding of a stronger association between BC and impaired cognition in individuals with longer TL is consistent with the hypothesis that leukocytes with longer TL mount stronger systemic inflammatory responses (Dioni et al. 2011; Hou et al. 2012). Increased systemic inflammatory responses to traffic-derived pollution may induce stronger effects on systemic targets (Fang et al. 2012), including the brain (Arfanakis et al. 2013). This hypothesis is particularly plausible because the stronger association between BC and cognition was found only in participants with TL in the highest quintile, whereas no dose–response relationship was found in participants with lower TL. This pattern of associations, which we also observed for the interaction between BC and CRP in this study, suggests the existence of an activation threshold, which is typical in inflammatory responses, particularly if they—such as those elicited by traffic air pollution—are induced through the NF-κB pathway (Nam et al. 2009). Of note, a previous study described an L-shaped association of TL with mild cognitive impairment; in particular, TL in the top quintile, but not in other quintiles, was associated with relative odds of both prevalent and incident mild cognitive impairment (Roberts et al. 2014). Alternatively, our finding might reflect a saturation effect among individuals with shorter TL. Those individuals may have experienced high levels of oxidative damage, they may have have higher background levels of oxidative stress, or they may have experienced a combination of the two. Because of this high oxidative state, BC exposure might only produce incremental oxidative damage in these individuals.

Inflammatory markers, including circulating CRP, have been associated with risk of dementia and cognitive decline in the elderly (Marioni et al. 2009). Neither TL nor MMSE score was significantly associated with CRP levels in the present study. However, we found that only higher CRP levels modified the association between BC exposure and cognitive function, supporting the hypothesis that traffic-related inflammatory response may induce stronger effects in systemic targets. To our knowledge, this is the first observation of the role of CRP as a modifier of the association between BC exposure and cognitive function in the elderly.

One limitation of this study is that it is based on relative TL rather than on absolute TL. However, previous comparisons show that qRT-PCR–measured relative TL correlates well with absolute TL (Ehrlenbach et al. 2009). Another limitation is that these findings are based on a cohort of older white men and may apply only to populations with similar characteristics. Furthermore, the VA NAS has a limited chronological age range (56–94 years), with 50% of participants between 69 and 78 years old. This limited age range may have contributed to the lack of association between TL and cognitive measures (Devore et al. 2011; Martin-Ruiz et al. 2006; Valdes et al. 2010; Yaffe et al. 2011). Finally, BC exposure was determined using geospatial models to estimate traffic-related pollution, and these estimates may differ from actual levels of personal exposure. However, as described previously, this approach is expected to produce nondifferential misclassification and is highly unlikely to bias results away from the null, but rather to underestimate the observed associations (Kioumourtzoglou et al. 2014). Furthermore, BC concentrations are highly spatially heterogeneous because of numerous local sources; therefore, the models used herein were expected to provide sufficient contrast.

## Conclusions

Our findings indicate that TL modifies the relationship between BC exposure and cognitive impairment observed in older men. High CRP levels were also predictive of increased susceptibility to cognitive impairment. Further research is warranted to confirm the findings of the present study in other study populations as well as to evaluate whether TL modifies the effects of BC exposure on other target systems, such as the cardiovascular and respiratory systems. These results increase our understanding of the role of biological factors in the response to environmental stressors in age-related cognitive impairment.

## Supplemental Material

(246 KB) PDF
