# Erratum: “Telomere Length, Long-Term Black Carbon Exposure, and Cognitive Function in a Cohort of Older Men: The VA Normative Aging Study”

**DOI:** 10.1289/EHP1813

**Published:** 2017-03-31

**Authors:** Elena Colicino, Ander Wilson, Maria Chiara Frisardi, Diddier Prada, Melinda C. Power, Mirjam Hoxha, Laura Dioni, Avron Spiro, Pantel S. Vokonas, Marc G. Weisskopf, Joel D. Schwartz, Andrea A. Baccarelli

Environ Health Perspect 125(1):76–81 (2017), http://dx.doi.org/10.1289/EHP241

In this erratum, the authors correct the information that appeared in their original article about the funding sources, including the agencies that provided the funding and the grant numbers:

This work was supported by the National Institute of Environmental Health Sciences (R01ES021733, R01ES015172, T32ES007142), the U.S. Environmental Protection Agency (RD83479801, RD832416, RD83587201), and the Agricultural Research Service of the U.S. Department of Agriculture (contract 53-K06-510). D. Prada received support from CONACYT and Fundación México en Harvard; M. Power received funding from the National Institute of Aging (T32 AG027668); and A. Spiro received support from the Clinical Science Research and Development Service of the U.S. Department of Veterans Affairs. The U.S. Department of Veterans Affairs (VA) Normative Aging Study (NAS) is supported by the Cooperative Studies Program and the Epidemiologic Research and Information Center (ERIC), which is a research component of the Massachusetts Veterans Epidemiology Research and Information Center (MAVERIC).

The views expressed in this paper are those of the authors and do not necessarily represent the views of the U.S. Department of VA.

The authors apologize for the missing information.

