# Supplementary material for: Telomere Length, Long-Term Black Carbon Exposure, and Cognitive Function in a Cohort of Older Men: The VA Normative Aging Study
Source: Environ Health Perspect. 2016 Jun 3;125(1):76–81. doi: 10.1289/EHP241 (PMC5226701; doi:10.1289/EHP241)
Supplement: (246 KB) PDF [file EHP241.s001.acco.pdf]

Environ Health Perspect
DOI: 10.1289/EHP241

Note to readers with disabilities: *EHP* strives to ensure that all journal content is accessible to all readers. However, some figures and Supplemental Material published in *EHP* articles may not conform to 508 standards due to the complexity of the information being presented. If you need assistance accessing journal content, please contact ehp508@niehs.nih.gov. Our staff will work with you to assess and meet your accessibility needs within 3 working days.

Supplemental Material

Telomere Length, Long-Term Black Carbon Exposure, and Cognitive Function in a Cohort of Older Men: The VA Normative Aging Study

Elena Colicino, Ander Wilson, Maria Chiara Frisardi, Diddier Prada, Melinda C. Power, Mirjam Hoxha, Laura Dioni, Avron Spiro III, Pantel S. Vokonas, Marc G. Weisskopf, Joel D. Schwartz, and Andrea A. Baccarelli

Table of Contents

Table S1. Eligible and non-eligible participants in NAS data between 1999 and 2007.

Table S2. Correlation coefficients between 1-Year BC exposure levels and BC exposure levels with different time windows.

Table S3. Demographic characteristics of NAS participants at first cognitive assessment on or after January 1, 1999. Mini-Mental State Examination (MMSE) scores, black carbon (BC) levels, telomere length (TL), and C-reactive protein (CRP) levels were reported.

Table S4. Distribution of NAS participants at the time of cognitive assessments by age in regards to Mini-Mental State Examination (MMSE) scores, black carbon (BC) levels, telomere length (TL), and C-reactive protein (CRP) levels.

Table S5. Relative Odds of low Mini-Mental State Examination (MMSE) score (≤25)[a] associated with C-reactive Protein (CRP) in quintiles.

Table S6. Sensitivity analysis. Relative odds of low Mini-Mental State Examination (MMSE) score (≤25)[a] associated with black carbon (BC) levels [b], by quintiles of telomere length (TL). MMSE outliers and CRP levels greater than 10 mg/L were excluded.

Table S7. Sensitivity analysis. Relative odds of low Mini-Mental State Examination (MMSE) score (≤25)[a] associated with black carbon (BC) levels [b], by quintiles of telomere length (TL) and age. MMSE outliers and CRP levels greater than 10 mg/L were excluded.

Table S8. Sensitivity analysis. Relative odds of low Mini-Mental State Examination (MMSE) score (≤25)[a] associated with black carbon (BC) levels [b], by quintiles of telomere length (TL) and C-reactive protein (CRP). MMSE outliers and CRP levels greater than 10 mg/L were excluded.

Table S9. Sensitivity analysis. Relative odds of low Mini-Mental State Examination (MMSE) score (≤25)[a] associated with black carbon (BC) levels [b], by quintiles of telomere length (TL). Not adjusting for hypertension, diabetes and coronary heart disease.

Table S10. Sensitivity analysis. Relative odds of low Mini-Mental State Examination (MMSE) score (≤25)[a] associated with black carbon (BC) levels [b], by quintiles of telomere length (TL) and age. Not adjusting for hypertension, diabetes and coronary heart disease.

Table S11. Sensitivity analysis. Relative odds of low Mini-Mental State Examination (MMSE) score (≤25)[a] associated with black carbon (BC) levels [b], by quintiles of telomere length (TL) and C-reactive protein (CRP). Not adjusting for hypertension, diabetes and coronary heart disease.

Table S1. Eligible and non-eligible participants in NAS data between 1999 and 2007.

Units Selected from Sampling Frame	Number of participants
**Respondent, total**	814
**Respondent not eligible**	
Experienced a stroke	36
**Eligible respondent with missing measures or data**	
Missing any cognitive information	95
Failed TL assay	46
Missing C-reactive protein information	10
Missing covariates	197
Missing BC estimates	2
**Eligible respondents with non-missing data for main analysis, total**	428
Outliers MMSE values	2
CRP measurements > 10 mg/L	8
**Eligible respondents for supplementary analysis, total**	418

Table S2. Correlation coefficients between 1-Year BC exposure levels and BC exposure levels with different time windows.

Exposure level	Pearson Correlation coefficients with BC 1-year	*p*	Number of observations
BC 1-year			622
BC 2-years	0.99	<.0001	597
BC 3-years	0.98	<.0001	495
BC 4-years	0.98	<.0001	386
BC 5-years	0.97	<.0001	211

Table S3. Demographic characteristics of NAS participants at first cognitive assessment on or after January 1, 1999. Mini-Mental State Examination (MMSE) scores, black carbon (BC) levels, telomere length (TL), and C-reactive protein (CRP) levels were reported.

Variable	N (%)	MMSE (≤25) N (%)	MMSE (>25) N (%)	BC Mean (StDev)	TL Mean (StDev)	CRP Mean (StDev)
All participants	428 (100%)	80 (18.69)	348 (81.31)	0.46 (0.23)	1.26 (0.51)	2.84 (5.71)
Age (years)						
[56–67]	96 (22.43)	8 (8.33)	88 (91.67)	0.48 (0.26)	1.42 (0.58)	3.61 (7.64)
[68–70]	64 (14.95)	10 (15.63)	54 (84.38)	0.42 (0.16)	1.26 (0.37)	2.55 (2.63)
[71–74]	113 (26.4)	14 (12.39)	99 (87.61)	0.46 (0.24)	1.21 (0.46)	1.99 (1.9)
[75–78]	55 (12.85)	10 (18.18)	45 (81.82)	0.4 (0.18)	1.11 (0.5)	4.53 (10.81)
[79–94]	100 (23.36)	38 (38)	62 (62)	0.49 (0.24)	1.22 (0.53)	2.31 (3.01)
Body mass index (kg/m^2)						
<25	88 (20.56)	16 (18.18)	72 (81.82)	0.47 (0.19)	1.23 (0.43)	1.69 (2.1)
≥25	340 (79.44)	64 (18.82)	276 (81.18)	0.46 (0.24)	1.26 (0.53)	3.13 (6.29)
Education (years)						
<12	124 (28.97)	38 (30.65)	86 (69.35)	0.48 (0.23)	1.19 (0.53)	2.8 (4.33)
12–16	211 (49.3)	28 (13.27)	183 (86.73)	0.46 (0.24)	1.28 (0.52)	3.15 (7.24)
>16	93 (21.73)	14 (15.05)	79 (84.95)	0.42 (0.20)	1.29 (0.44)	2.17 (2.42)
Computer experience						
No	204 (47.66)	23 (11.27)	181 (88.73)	0.44 (0.2)	1.29 (0.53)	2.71 (5.71)
Yes	224 (52.34)	57 (25.45)	167 (74.55)	0.47 (0.25)	1.22 (0.48)	2.95 (5.72)
Physical activity (MET-hr/week)						
<12	275 (64.25)	60 (21.82)	215 (78.18)	0.45 (0.23)	1.24 (0.47)	3.22 (6.94)
12–30	97 (22.66)	14 (14.43)	83 (85.57)	0.48 (0.25)	1.3 (0.6)	2.3 (2.11)
≥30	56 (13.08)	6 (10.71)	50 (89.29)	0.46 (0.21)	1.28 (0.5)	1.88 (1.87)
First language						
English	365 (85.28)	60 (16.44)	305 (83.56)	0.46 (0.23)	1.27 (0.51)	2.61 (4.67)
Not English	63 (14.72)	20 (31.75)	43 (68.25)	0.44 (0.2)	1.2 (0.51)	4.13 (9.72)

Dark-meat fish consumption						
<1	371 (86.68)	76 (20.49)	295 (79.51)	0.45 (0.23)	1.27 (0.52)	2.98 (6.07)
≥1	57 (13.32)	4 (7.02)	53 (92.98)	0.49 (0.22)	1.19 (0.43)	1.92 (2.1)
% census tract that is nonwhite						
<5%	168 (39.25)	28 (16.67)	140 (83.33)	0.42 (0.26)	1.24 (0.52)	3.07 (6.22)
5–10%	113 (26.4)	20 (17.7)	93 (82.3)	0.43 (0.21)	1.28 (0.52)	2.98 (7.09)
>10%	147 (34.35)	32 (21.77)	115 (78.23)	0.52 (0.2)	1.25 (0.49)	2.46 (3.51)
% census tract (≥25-years-old) with at least a college degree						
<30%	134 (31.31)	29 (21.64)	105 (78.36)	0.49 (0.23)	1.23 (0.52)	2.9 (4.05)
30–50%	171 (39.95)	27 (15.79)	144 (84.21)	0.44 (0.26)	1.26 (0.52)	3.19 (7.84)
≥50%	123 (28.74)	24 (19.51)	99 (80.49)	0.46 (0.18)	1.29 (0.49)	2.27 (3.18)
Alcohol (drinks/day)						
<2	335 (78.27)	64 (19.1)	271 (80.9)	0.46 (0.24)	1.26 (0.51)	2.6 (3.64)
≥2	93 (21.73)	16 (17.2)	77 (82.8)	0.44 (0.17)	1.22 (0.49)	3.69 (10.11)
Smoking						
Never	120 (28.04)	23 (19.17)	97 (80.83)	0.45 (0.22)	1.24 (0.48)	3.21 (7.23)
Current	13 (3.04)	2 (15.38)	11 (84.62)	0.46 (0.15)	1.16 (0.33)	2.97 (2.35)
Former	295 (68.93)	55 (18.64)	240 (81.36)	0.46 (0.24)	1.27 (0.53)	2.68 (5.09)
Diabetes						
No	352 (82.24)	60 (17.05)	292 (82.95)	0.46 (0.24)	1.26 (0.51)	2.71 (5.07)
Yes	76 (17.76)	20 (26.32)	56 (73.68)	0.43 (0.18)	1.23 (0.49)	3.41 (8.05)
Hypertension						
No	130 (30.37)	19 (14.62)	111 (85.38)	0.42 (0.23)	1.23 (0.49)	2.77 (6.49)
Yes	298 (69.63)	61 (20.47)	237 (79.53)	0.48 (0.23)	1.27 (0.51)	2.86 (5.35)
Coronary heart disease						
No	303 (70.79)	57 (18.81)	246 (81.19)	0.44 (0.2)	1.27 (0.49)	2.82 (6.17)
Yes	125 (29.21)	23 (18.4)	102 (81.6)	0.5 (0.28)	1.23 (0.55)	2.88 (4.43)

Table S4. Distribution of NAS participants at the time of cognitive assessments by age in regards to Mini-Mental State Examination (MMSE) scores, black carbon (BC) levels, telomere length (TL), and C-reactive protein (CRP) levels.

Variable	N (%)	MMSE (≤25) N (%)	MMSE (>25) N (%)	BC Mean (StDev)	TL Mean (StDev)	CRP Mean (StDev)
Participants at first visit	428 (100.00)	80 (18.69)	348 (81.31)	0.46 (0.23)	1.26 (0.51)	2.84 (5.71)
Age (years)						
[56–67]	96 (22.43)	8 (8.33)	88 (91.67)	0.48 (0.26)	1.42 (0.58)	3.61 (7.64)
[68–70]	64 (14.95)	10 (15.63)	54 (84.38)	0.42 (0.16)	1.26 (0.37)	2.55 (2.63)
[71–74]	113 (26.4)	14 (12.39)	99 (87.61)	0.46 (0.24)	1.21 (0.46)	1.99 (1.90)
[75–78]	55 (12.85)	10 (18.18)	45 (81.82)	0.40 (0.18)	1.11 (0.50)	4.53 (10.81)
[79–94]	100 (23.36)	38 (38.00)	62 (62.00)	0.49 (0.24)	1.22 (0.53)	2.31 (3.01)
Participants at second visit	173 (100.00)	38 (21.97)	135 (78.03)	0.41 (0.20)	1.14 (0.49)	3.26 (4.05)
Age (years)						
[56–67]	17 (9.83)	4 (23.53)	13 (76.47)	0.43 (0.28)	1.38 (0.47)	3.39 (4.81)
[68–70]	21 (12.14)	3 (14.29)	18 (85.71)	0.42 (0.21)	1.06 (0.42)	4.49 (6.76)
[71–74]	45 (26.01)	9 (20.00)	36 (80.00)	0.42 (0.16)	1.11 (0.41)	3.91 (4.67)
[75–78]	50 (28.90)	9 (18.00)	41 (82.00)	0.38 (0.21)	1.14 (0.57)	2.43 (2.26)
[79–94]	40 (23.12)	13 (32.50)	27 (67.50)	0.42 (0.21)	1.12 (0.48)	2.89 (2.47)
Participants at third visit	21 (100.00)	6 (28.57)	15 (71.43)	0.39 (0.22)	1.07 (0.42)	2.1 (1.67)
Age (years)						
[56–67]	2 (9.52)	1 (50.00)	1 (50.00)	0.54 (0.20)	1.41 (0.38)	0.58 (0.43)
[68–70]	2 (9.52)	1 (50.00)	1 (50.00)	0.57 (0.27)	1.7 (0.52)	1.58 (1.08)
[71–74]	5 (23.81)	1 (20.00)	4 (80.00)	0.38 (0.15)	0.72 (0.16)	2.4 (1.77)
[75–78]	4 (19.05)	0 (0.00)	4 (100.00)	0.35 (0.21)	1.05 (0.53)	1.59 (0.69)
[79–94]	8 (38.10)	3 (37.50)	5 (62.50)	0.32 (0.25)	1.05 (0.29)	2.69 (2.11)

Table S5. Relative Odds of low Mini-Mental State Examination (MMSE) score (≤25)[a] associated with C-reactive Protein (CRP) in quintiles.

Association between BC and low MMSE (≤25) by CRP	OR	95% CI	*p*	Cases	Non-cases
CRP 1st quintile (0.04-0.66)	Ref.	.	.	24	99
CRP 2nd quintile (0.67-1.20)	0.79	(0.39, 1.59)	0.51	23	102
CRP 3rd quintile (1.21-2.04)	1.06	(0.53, 2.13)	0.86	32	92
CRP 4th quintile (2.04-3.99)	0.83	(0.42, 1.66)	0.60	24	100
CRP 5th quintile (4.00-72.20)	0.68	(0.34, 1.36)	0.28	21	105

OR=Odds Ratio, 95% CI=95% Confidence interval.

[a]Adjusted for age, education level, first language, computer experience, physical activity level, body mass index, dark fish consumption, alcohol consumption, smoking status, percentage of adults with a college degree, percentage of the participant's census tract that is nonwhite, indicator for first cognitive assessment, indicator for part-time resident, hypertension, diabetes, coronary heart disease, and telomere measurements.

Table S6. Sensitivity analysis. Relative odds of low Mini-Mental State Examination (MMSE) score (≤25)[a] associated with black carbon (BC) levels [b], by quintiles of telomere length (TL). MMSE outliers and CRP levels greater than 10 mg/L were excluded.

Association between BC and low MMSE (≤25) by TL	OR for BC	95% CI	*p*	*p* for interaction[c]	Cases	Non-cases
TL 1st quintile (0.30–0.79)	1.25	(0.81, 1.91)	0.31	.	26	94
TL 2nd quintile (0.80–1.04)	1.46	(0.96, 2.23)	0.08	0.59	23	96
TL 3rd quintile (1.05–1.25)	1.71	(0.86, 3.39)	0.13	0.45	21	100
TL 4th quintile (1.26–1.56)	1.06	(0.63, 1.77)	0.84	0.61	26	94
TL 5th quintile (1.57–3.81)	3.90	(1.50, 10.16)	0.01	0.03	21	99
All interaction terms at once (Wald test)				0.03	117	483

OR=odds ratio; 95% CI=95% confidence interval

[a] Adjusted for education level, first language, computer experience, physical activity level, body mass index, dark-meat fish consumption, alcohol consumption, smoking status, percent of adults with a college degree, percentage of the participant's census tract that is nonwhite, indicator for first cognitive assessment, indicator for part-time resident, hypertension, diabetes, coronary heart disease, and C-reactive protein levels.

[b] Effect of each doubling in BC level corresponding to a 0.69 $\mu g/m^3$ increase in average ln(BC) concentration

[c] TL by BC level interaction

Table S7. Sensitivity analysis. Relative odds of low Mini-Mental State Examination (MMSE) score (≤25)[a] associated with black carbon (BC) levels [b], by quintiles of telomere length (TL) and age. MMSE outliers and CRP levels greater than 10 mg/L were excluded.

**Association between BC and low MMSE (≤25) by TL or Age**	**OR for BC**	**95% CI**	***p***	***p* for interaction**	**Cases**	**Non-cases**
TL 1st quintile (0.30–0.79)	1.10	(0.56, 2.14)	0.80	.	26	94
TL 2nd quintile (0.80–1.04)	1.32	(0.71, 2.44)	0.38	0.54 [c]	23	96
TL 3rd quintile (1.05–1.25)	1.46	(0.67, 3.19)	0.35	0.48 [c]	21	100
TL 4th quintile (1.26–1.56)	0.93	(0.42, 2.07)	0.86	0.65 [c]	26	94
TL 5th quintile (1.57–3.81)	3.01	(1.16, 7.81)	0.02	0.02 [c]	21	99
Age 1st quintile (56–67)	1.55	(0.53, 4.56)	0.43	0.50 [d]	12	96
Age 2nd quintile (68–70)	1.85	(0.56, 6.16)	0.32	0.30 [d]	13	71
Age 3rd quintile (71–74)	0.90	(0.38, 2.11)	0.82	0.51 [d]	23	138
Age 4th quintile (75–78)	1.30	(0.55, 3.10)	0.57	0.66 [d]	18	86
Age 5th quintile (79–94)	1.10	(0.56, 2.14)	0.80	.	51	92
All interaction terms at once (Wald test)				0.10	117	483

OR=odds ratio; 95% CI=95% confidence interval

[a] Adjusted for education level, first language, computer experience, physical activity level, body mass index, dark-meat fish consumption, alcohol consumption, smoking status, percent of adults with a college degree, percentage of the participant's census tract that is nonwhite, indicator for first cognitive assessment, indicator for part-time resident, hypertension, diabetes, coronary heart disease, and C-reactive protein levels.

[b] Effect of each doubling in BC level corresponding to a 0.69 $\mu g/m^3$ increase in average ln(BC) concentration

[c] *p*-values for TL by BC level interaction

[d] *p*-values for age by BC level interaction

Table S8. Sensitivity analysis. Relative odds of low Mini-Mental State Examination (MMSE) score (≤25)[a] associated with black carbon (BC) levels[b], by quintiles of telomere length (TL) and C-reactive protein (CRP). MMSE outliers and CRP levels greater than 10 mg/L were excluded.

Association between BC and low MMSE (≤25) by TL or CRP	OR	95% CI	*p*	*p* for interaction	Cases	Non-cases
TL 1st quintile (0.30–0.79)	0.72	(0.33, 1.60)	0.43	.	26	94
TL 2nd quintile (0.80–1.04)	1.05	(0.43, 2.58)	0.92	0.28[c]	23	96
TL 3rd quintile (1.05–1.25)	1.16	(0.43, 3.10)	0.78	0.36[c]	21	100
TL 4th quintile (1.26–1.56)	0.75	(0.28, 2.02)	0.58	0.92[c]	26	94
TL 5th quintile (1.57–3.81)	2.60	(0.85, 7.89)	0.09	0.02[c]	21	99
CRP 1st quintile (0.04–0.66)	0.72	(0.33, 1.60)	0.43	.	22	94
CRP 2nd quintile (0.67–1.20)	1.45	(0.67, 3.15)	0.35	0.16[d]	23	96
CRP 3rd quintile (1.21–2.04)	0.99	(0.34, 2.90)	0.99	0.41[d]	28	100
CRP 4th quintile (2.04–3.99)	0.62	(0.20, 1.96)	0.43	0.77[d]	24	94
CRP 5th quintile (4.00–72.20)	2.97	(1.15, 7.67)	0.02	0.03[d]	20	99
All interaction terms at once (Wald test)				0.06	117	483

OR=odds ratio; 95% CI=95% confidence interval

[a] Adjusted for education level, first language, computer experience, physical activity level, body mass index, dark-meat fish consumption, alcohol consumption, smoking status, percent of adults with a college degree, percentage of the participant’s census tract that is nonwhite, indicator for first cognitive assessment, indicator for part-time resident, hypertension, diabetes and coronary heart disease.

[b] Effect of each doubling in BC level corresponding to a 0.69 μg/m^3 increase in average ln(BC) concentration

[c] *p*-value for TL by BC level interaction

[d] *p*-value for CRP level by BC level interaction

Table S9. Sensitivity analysis. Relative odds of low Mini-Mental State Examination (MMSE) score (≤25)[a] associated with black carbon (BC) levels [b], by quintiles of telomere length (TL). Not adjusting for hypertension, diabetes and coronary heart disease.

**Association between BC and low MMSE (≤25) by TL**	**OR for BC**	**95% CI**	***p***	***p* for interaction**[c]	**Cases**	**Non-cases**
TL 1st quintile (0.30–0.79)	1.27	(0.83, 1.93)	0.27		28	96
TL 2nd quintile (0.80–1.04)	1.38	(0.94, 2.02)	0.10	0.76	25	99
TL 3rd quintile (1.05–1.25)	1.76	(0.86, 3.60)	0.12	0.44	22	103
TL 4th quintile (1.26–1.56)	1.07	(0.65, 1.77)	0.78	0.60	27	97
TL 5th quintile (1.57–3.81)	2.86	(1.26, 6.49)	0.01	0.07	22	103
All interaction terms at once (Wald test)				0.60	124	498

OR=odds ratio; 95% CI=95% confidence interval

[a] Adjusted for education level, first language, computer experience, physical activity level, body mass index, dark-meat fish consumption, alcohol consumption, smoking status, percent of adults with a college degree, percentage of the participant's census tract that is nonwhite, indicator for first cognitive assessment, indicator for part-time resident and C-reactive protein levels.

[b] Effect of each doubling in BC level corresponding to a 0.69 $\mu g/m^3$ increase in average ln(BC) concentration

[c] TL by BC level interaction

Table S10. Sensitivity analysis. Relative odds of low Mini-Mental State Examination (MMSE) score (≤25)[a] associated with black carbon (BC) levels [b], by quintiles of telomere length (TL) and age. Not adjusting for hypertension, diabetes and coronary heart disease.

Association between BC and low MMSE (≤25) by TL or Age	OR for BC	95% CI	*p*	*p* for interaction	Cases	Non-cases
TL 1st quintile (0.30–0.79)	1.04	(0.54, 1.99)	0.91	.	28	96
TL 2nd quintile (0.80–1.04)	1.22	(0.67, 2.23)	0.52	0.548[c]	25	99
TL 3rd quintile (1.05–1.25)	1.51	(0.69, 3.31)	0.30	0.37 [c]	22	103
TL 4th quintile (1.26–1.56)	0.90	(0.42, 1.94)	0.80	0.69 [c]	27	97
TL 5th quintile (1.57–3.81)	2.20	(0.98, 4.93)	0.06	0.06 [c]	22	103
Age 1st quintile (56–67)	1.37	(0.49, 3.83)	0.56	0.546[d]	13	102
Age 2nd quintile (68–70)	1.66	(0.53, 5.21)	0.39	0.34 [d]	14	73
Age 3rd quintile (71–74)	0.94	(0.41, 2.12)	0.88	0.71 [d]	24	139
Age 4th quintile (75–78)	1.60	(0.67, 3.82)	0.29	0.29 [d]	19	90
Age 5th quintile (79–94)	1.04	(0.54, 1.99)	0.91	.	54	94
All interaction terms at once (Wald test)				0.18	124	498

OR=odds ratio; 95% CI=95% confidence interval

[a] Adjusted for education level, first language, computer experience, physical activity level, body mass index, dark-meat fish consumption, alcohol consumption, smoking status, percent of adults with a college degree, percentage of the participant's census tract that is nonwhite, indicator for first cognitive assessment, indicator for part-time resident and C-reactive protein levels.

[b] Effect of each doubling in BC level corresponding to a 0.69 $\mu g/m^3$ increase in average ln(BC) concentration

[c] *p*-values for TL by BC level interaction

[d] *p*-values for age by BC level interaction

Table S11. Sensitivity analysis. Relative odds of low Mini-Mental State Examination (MMSE) score (≤25)[a] associated with black carbon (BC) levels [b], by quintiles of telomere length (TL) and C-reactive protein (CRP). Not adjusting for hypertension, diabetes and coronary heart disease.

Association between BC and low MMSE (≤25) by TL or CRP	OR	95% CI	*p*	*p* for interaction	Cases	Non-cases
TL 1st quintile (0.30–0.79)	0.77	(0.36, 1.65)	0.51	.	28	96
TL 2nd quintile (0.80–1.04)	0.98	(0.42, 2.31)	0.97	0.4634	25	99
TL 3rd quintile (1.05–1.25)	1.24	(0.47, 3.25)	0.68	0.3435	22	103
TL 4th quintile (1.26–1.56)	0.79	(0.30, 2.05)	0.64	0.9473	27	97
TL 5th quintile (1.57–3.81)	1.86	(0.69, 5.06)	0.23	0.05	22	103
CRP 1st quintile (0.04–0.66)	0.77	(0.36, 1.65)	0.51	.	24	99
CRP 2nd quintile (0.67–1.20)	1.52	(0.72, 3.19)	0.28	0.1584	23	102
CRP 3rd quintile (1.21–2.04)	1.03	(0.36, 2.94)	0.96	0.4177	32	92
CRP 4th quintile (2.04–3.99)	0.73	(0.26, 2.07)	0.57	0.9236	24	100
CRP 5th quintile (4.00–72.20)	2.64	(1.06, 6.61)	0.04	0.0346	21	105
All interaction terms at once (Wald test)				0.1	124	498

OR=odds ratio; 95% CI=95% confidence interval

[a] Adjusted for education level, first language, computer experience, physical activity level, body mass index, dark-meat fish consumption, alcohol consumption, smoking status, percent of adults with a college degree, percentage of the participant's census tract that is nonwhite, indicator for first cognitive assessment and indicator for part-time resident.

[b] Effect of each doubling in BC level corresponding to a 0.69 μg/m^3 increase in average ln(BC) concentration

[c] *p*-values for TL by BC level interaction

[d] *p*-values for CRP by BC level interaction
